# Transmembrane emp24 domain-containing protein 3 promotes the malignant progression of glioma by regulating the ZBTB7A signaling axis

**DOI:** 10.1186/s43556-025-00274-7

**Published:** 2025-06-05

**Authors:** Yang Qiao, Lv Zhou, Jianyu Nie, Jinshui Li, Yangchun Hu, Peng Gao, Bingshan Wu, Hongwei Cheng, Xingliang Dai

**Affiliations:** 1 Department of Neurosurgery, the First Affiliated Hospital of Anhui Medical University, Hefei, Anhui 230001 China; 2 Department of Clinical Medicine, the Second Clinical College of Anhui Medical University, Hefei, Anhui 230032 China

**Keywords:** Transmembrane emp24 domain-containing protein 3 (TMED3), Zinc finger and BTB domain-containing protein 7A (ZBTB7 A), Single-Cell Sequencing, Glioblastoma (GBM), Target therapy

## Abstract

Glioblastoma (GBM) is highly malignant with a poor prognosis. Exploring new therapeutic targets in GBM is an effective strategy for the prognosis of GBM patients. The Transmembrane emp24 domain-containing protein 3 (TMED3) gene has been found to play a role in the development of various cancers, but its mechanism in GBM remains unclear. This study combined the TCGA database, single-cell RNA sequencing, and in vitro and in vivo experiments to systematically investigate the role of TMED3 in GBM and its potential mechanisms. The study found that the TMED3 gene is differentially expressed in GBM samples, and high expression is associated with a higher grade of GBM and a poorer prognosis. In vitro and in vivo experiments confirmed that the upregulation of TMED3 promoted GBM proliferation, invasion, and migration. Further immunoprecipitation and functional rescue experiments revealed that Zinc finger and BTB domain-containing protein 7A (ZBTB7A) acts as a downstream target of TMED3. TMED3 promotes the malignant progression of GBM by regulating ZBTB7A. In conclusion, this study reveals that TMED3 promotes GBM development through the regulation of the ZBTB7A signaling axis, providing new insights for targeted therapy of GBM.

**Supplementary Information:**

The online version contains supplementary material available at 10.1186/s43556-025-00274-7.

## Introduction

Glioma, the most prevalent and aggressive primary brain tumor, is characterized by high incidence, extensive invasiveness, frequent recurrence, and poor prognosis, particularly in glioblastoma (GBM). The median survival time for GBM patients is typically less than 15 months [1–3]. Despite advancements in surgery, radiotherapy, chemotherapy, and molecular-targeted therapies, overall therapeutic efficacy remains limited, and treatment outcomes continue to pose significant challenges [4–6]. The complex molecular mechanisms underlying GBM are considered key factors limiting treatment success [7]. These mechanisms include genetic alterations, such as IDH mutations and TERT promoter mutations, as well as dysregulated signaling pathways, including the PI3 K/AKT/mTOR axis and EGFR amplification. These abnormalities directly influence tumor cell metabolism, growth, migration, and invasion, while also correlating with the prognosis of patients [8–11]. Therefore, elucidating the molecular mechanisms of GBM, particularly its key regulatory molecules and signaling pathways, is critical. Such insights may reveal the drivers of tumor progression and provide novel therapeutic strategies to improve patient outcomes.

Cancer-related genes such as Transmembrane emp24 domain-containing protein 3 (TMED3) have gained widespread attention due to their roles in various types of cancer [12]. TMED3, a member of the p24 family, encodes a transmembrane protein involved in trafficking between the endoplasmic reticulum and the Golgi complex [13]. TMED3 plays a critical role in maintaining cellular homeostasis, including protein folding, secretion, and membrane protein transport. Recent research has emphasized the dual role of TMED3 in various cancers. In some tumors, abnormal expression of TMED3 promotes tumor cell growth, migration, and invasion by regulating key signaling axes such as Wnt/β-catenin and ERK/MAPK. For instance, elevated TMED3 expression in osteosarcoma and breast cancer is closely linked to enhanced tumor invasiveness and a worse prognosis [14, 15]. However, in other cases, TMED3 may exhibit a protective effect by regulating epithelial-mesenchymal transition or cell adhesion molecules to inhibit the spread of tumor cells [16]. While the precise role of TMED3 in different cancers is not fully understood, its complex involvement in tumor progression makes it an important target for studying tumor mechanisms and potential therapeutic strategies.

Single-cell sequencing technology offers an in-depth analysis of genomes, transcriptomes, and epigenomes at the single-cell level, providing valuable insights into the complex molecular mechanisms of gliomas [17, 18]. Through single-cell sequencing, we can gain a clearer understanding of TMED3's function in different glioma cell subpopulations [19, 20]. Additionally, bioinformatics analysis—integrating gene expression data, clinical information, and public glioma databases—allows for the systematic exploration of these complex biological datasets, revealing the involvement of TMED3 in critical biological functions, including tumor cell growth, migration, and resistance to therapy [21–23]. Techniques like immunoprecipitation, gene co-expression network construction, and gene knockdown experiments can further validate TMED3's function and identify potential downstream regulatory factors [24]. The integrated application of these technologies helps to deepen our understanding of the role of TMED3 in gliomas, providing a theoretical foundation and experimental support for the development of targeted TMED3 therapeutic strategies.

In this study, we systematically investigated TMED3's role in GBM using single-cell sequencing and bioinformatics analysis. Our results showed that TMED3 is highly expressed in GBM samples, and its overexpression correlates with higher tumor grade and poor prognosis. Using an in vitro TMED3 knockdown model, we assessed the impact of TMED3 on key malignant phenotypes of glioma cells, including proliferation, invasion, migration, and apoptosis. Our experiments demonstrated that overexpression of TMED3 significantly enhanced GBM cell proliferation, invasion, and migration. In vivo experiments further confirmed that high TMED3 expression promotes glioma initiation and progression. Through co-immunoprecipitation (Co-IP) proteomics, we identified Zinc finger and BTB domain-containing protein 7 A (ZBTB7A) as a direct interacting partner of TMED3. Functional rescue experiments confirmed that TMED3 promotes GBM cell growth, migration, and invasion by directly regulating ZBTB7A expression. In conclusion, this study reveals the crucial role of TMED3 in the initiation and progression of gliomas and uncovers its potential molecular mechanisms. These findings deepen our understanding of the mechanisms driving malignant progression in gliomas, offering novel ideas for the development of future therapeutic strategies.

## Results

### TMED3 is significantly differentially expressed in GBM specimens and associated with a higher GBM grade and a poorer prognosis

**Fig. 1:**
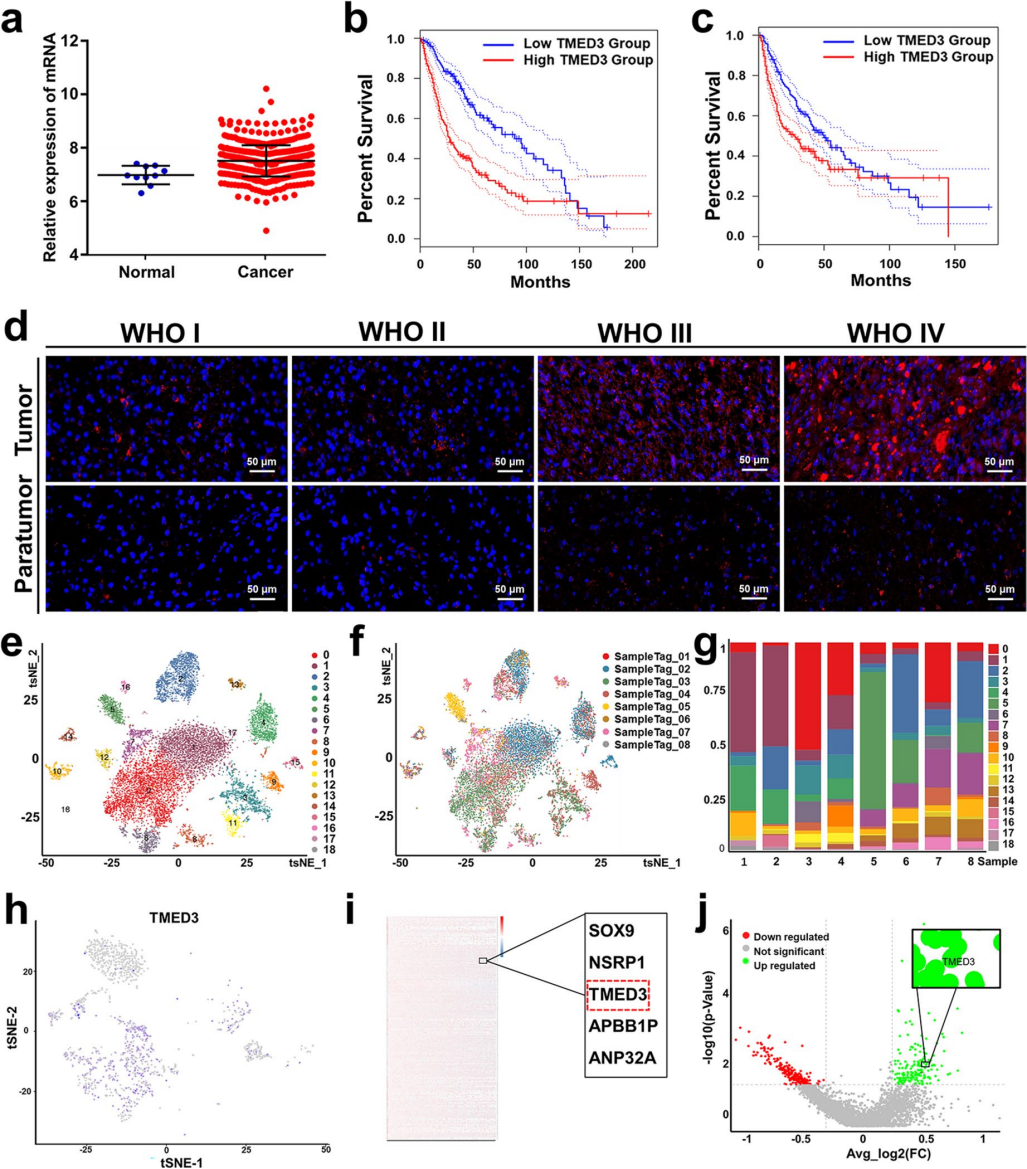
TMED3 is differentially expressed in GBM and associated with higher grades and poor prognosis. **a** Expression levels of TMED3 in glioma and normal tissues. **b** OS of glioma patients with different TMED3 expression levels. **c** DFS of glioma patients with different TMED3 expression levels. **d** Representative immunofluorescence staining of TMED3 in glioma samples of different grades. **e** Cell subpopulations in GBM samples (19 clusters). **f** Distribution of cell subpopulations across different GBM samples. **g** Ratio of cell subpopulations in various samples. **h** The expression and distribution of TMED3 in glial cell subpopulation. **i-j** Heatmap and volcano plot comparing glioma cluster 0 and normal tissues

To investigate the role of TMED3 in GBM, we first performed bioinformatics analysis using GBM data from the TCGA database. Initially, we analyzed the significance of differences in TMED3 expression levels across various clinical samples. The results revealed a significant difference in TMED3 expression between normal and tumor tissues, suggesting a potential correlation between TMED3 expression and these clinical samples (Fig. 1a). Furthermore, we examined whether different TMED3 expression levels impacted patient survival outcomes (Tab S1, S2). The results demonstrated that TMED3 expression levels significantly affected patient survival, with GBM patients exhibiting higher TMED3 expression showing markedly reduced overall survival (OS) and disease-free survival (DFS) in comparison to individuals with low TMED3 expression (Fig. 1b, c). Additionally, immunofluorescence analysis of GBM and adjacent normal tissues revealed that TMED3 expression was higher in GBM tissues than in normal brain tissues, and its expression increased with higher GBM grades (Fig. 1d). These findings preliminarily indicate that TMED3 is significantly expressed in GBM, and its high expression is linked to higher GBM grades and a poorer prognosis.

To further validate the expression of TMED3 in GBM, we performed single-cell RNA sequencing on four GBM samples and four adjacent normal tissue samples. A total of 12,118 cells were isolated, with each cell detecting approximately 3,093 genes and 11,178 UMIs. The cell subpopulations within the GBM microenvironment were classified into 19 clusters (Fig. 1e). The expression profiles of cell subpopulations across different samples are shown in Fig. 1f, g. Based on cell type-specific markers, these clusters were annotated as CD45^+^ immune cells (clusters 0, 1, 4, 6, 7, 8, 12, 13), including monocytes (clusters 0, 1, 6, 7) and CD3^+^ T cells (clusters 4, 13). More detailed cell subpopulation annotations are provided in Fig. S1. Among the 19 clusters, 10 distinct cell subtypes were identified: monocytes (51.5%), astrocytes (19.7%), oligodendrocytes (11.0%), T cells (9.1%), mesenchymal stromal cells (2.4%), endothelial cells (1.4%), dendritic cells (1.8%), neural progenitor cells (1.3%), B lymphocytes (0.6%), and unclassified cells (1.0%). We ranked the representative genes for each subpopulation based on confidence levels and visualized their expression patterns across tSNE clusters, with gray indicating low expression and darker blue indicating higher expression levels (Fig. S2). Subsequently, we re-clustered and analyzed the glial cell subpopulation. The re-clustering resulted in 11 clusters, and the distribution of TMED3 across these 11 clusters is shown in Fig. 1h. Among them, the most significant differential expression of TMED3 between normal and tumor tissues was observed in glioma cluster 0 (Fig. S3). This cluster was characterized by markers such as apolipoprotein D (APOD) and myelin-associated glycoprotein (MAG) (Fig. S4). Further comparative analysis of glioma cluster 0 with adjacent normal tissues highlighted TMED3 as a differentially expressed gene in GBM, as shown in the heatmap and volcano plot analyses (Fig. 1i, j). Based on these bioinformatics results, we conclude that TMED3 is differentially expressed in GBM cells, particularly in specific subpopulations, and its high expression is closely associated with higher GBM grades and poor prognosis.

### The knockdown of TMED3 inhibits GBM cell proliferation, migration, and invasion

**Fig. 2:**
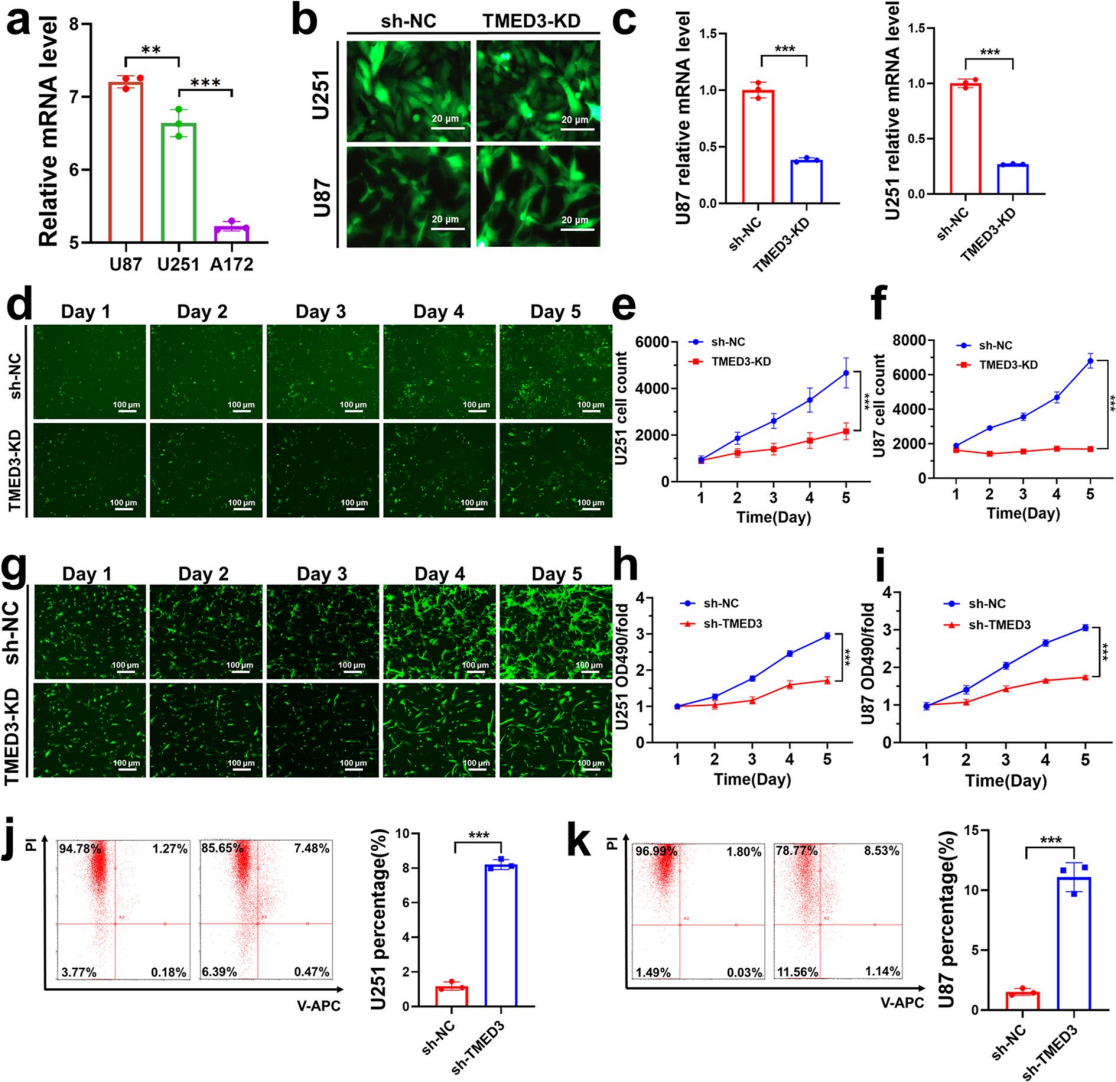
In vitro validation of the effects of TMED3 on GBM cell proliferation and apoptosis. **a** the expression levels of TMED3 in three glioma cell lines. **b**, **c** TMED3 knockdown efficiency and statistical analysis after lentiviral transfection. **d–g** Celigo cell count results and statistical analysis for U251 (**d**, **e**) and U87 (**f**,** g**) cells. **h**, **i** MTT assay results for U251 and U87 cells after TMED3-KD transfection. **j**, **k** Apoptosis assay results and analysis for U251 and U87 cells after TMED3-KD transfection. (*n* = 3, *P < 0.05, **P < 0.01, ***P < 0.001)

Based on bioinformatics analysis and single-cell sequencing validation, we preliminarily confirmed that TMED3 is differentially expressed in GBM, and its high expression is closely associated with higher GBM grades and poorer prognosis. To further explore the role of TMED3 in GBM cells, we conducted in-depth in vitro experiments. First, we assessed TMED3 expression levels in three common GBM cell lines using qPCR to select suitable cell lines for subsequent experiments. The results indicated that TMED3 expression was highest in U251 and U87 cells (Fig. 2a). Therefore, these two cell lines were chosen for lentivirus-mediated TMED3 knockdown experiments. The knockdown efficiency of TMED3 was evaluated at the mRNA level. At 72 h post-infection, fluorescence microscopy revealed that the infection efficiency reached 80% in both U87 and U251 cells, with no significant morphological abnormalities observed (Fig. 2b, c). TMED3 knockdown significantly reduced TMED3 mRNA expression in U87 and U251 cells (*P* < 0.05), with knockdown efficiencies of 73.1% and 61.5%, respectively. These results confirmed that lentiviral transfection effectively suppressed TMED3 mRNA expression.

**Fig. 3:**
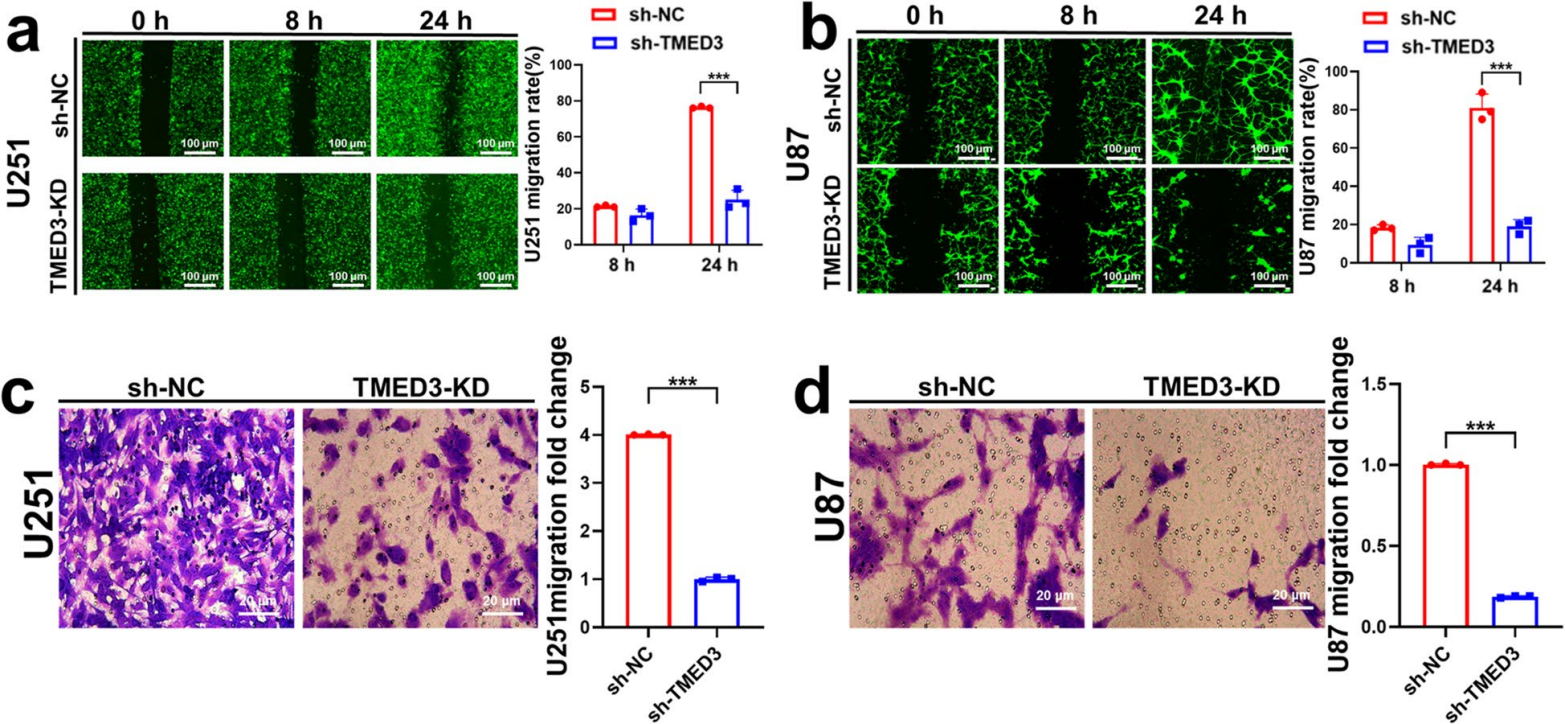
In vitro validation of the effects of TMED3 on GBM cell migration and invasion. **a**,** b** Wound healing assay results and quantitative analysis for U251 and U87 cells. **c**,** d** Transwell migration assay results and statistical analysis for U251 and U87 cells. (*n* = 3, *P < 0.05, **P < 0.01, ***P < 0.001)

Next, we validated the impact of TMED3 knockdown on GBM cells. Cell proliferation assays demonstrated that silencing TMED3 notably reduced the growth of U251 and U87 cells compared to the control group, as shown by Celigo cell counting (Fig. 2d-g). This finding was corroborated by MTT assays, which also revealed a marked reduction in proliferation rates in TMED3-knockdown U251 and U87 cells (Fig. 2h, i). Additionally, apoptosis assays indicated that by day 5 post-transfection, the apoptosis rate was considerably greater in the TMED3-knockdown group compared to the control group for both U251 and U87 cells (Fig. 2j, k). To investigate the impact of TMED3 on GBM cell migration, we performed wound healing and transwell assays. The wound healing assay demonstrated that TMED3 knockdown significantly reduced the migration rate of U251 and U87 cells at 24 h (Fig. 3a, b). Similarly, transwell assays showed a notable decrease in the number of U251 and U87 cells migrating through the transwell chamber in the TMED3-knockdown group compared to controls (Fig. 3c, d). In summary, these results indicate that TMED3 knockdown significantly inhibits GBM cell proliferation, migration, and invasion in vitro, highlighting the potential of TMED3 as a therapeutic target in GBM.

### TMED3 knockdown inhibits tumorigenic effects in mice

**Fig. 4:**
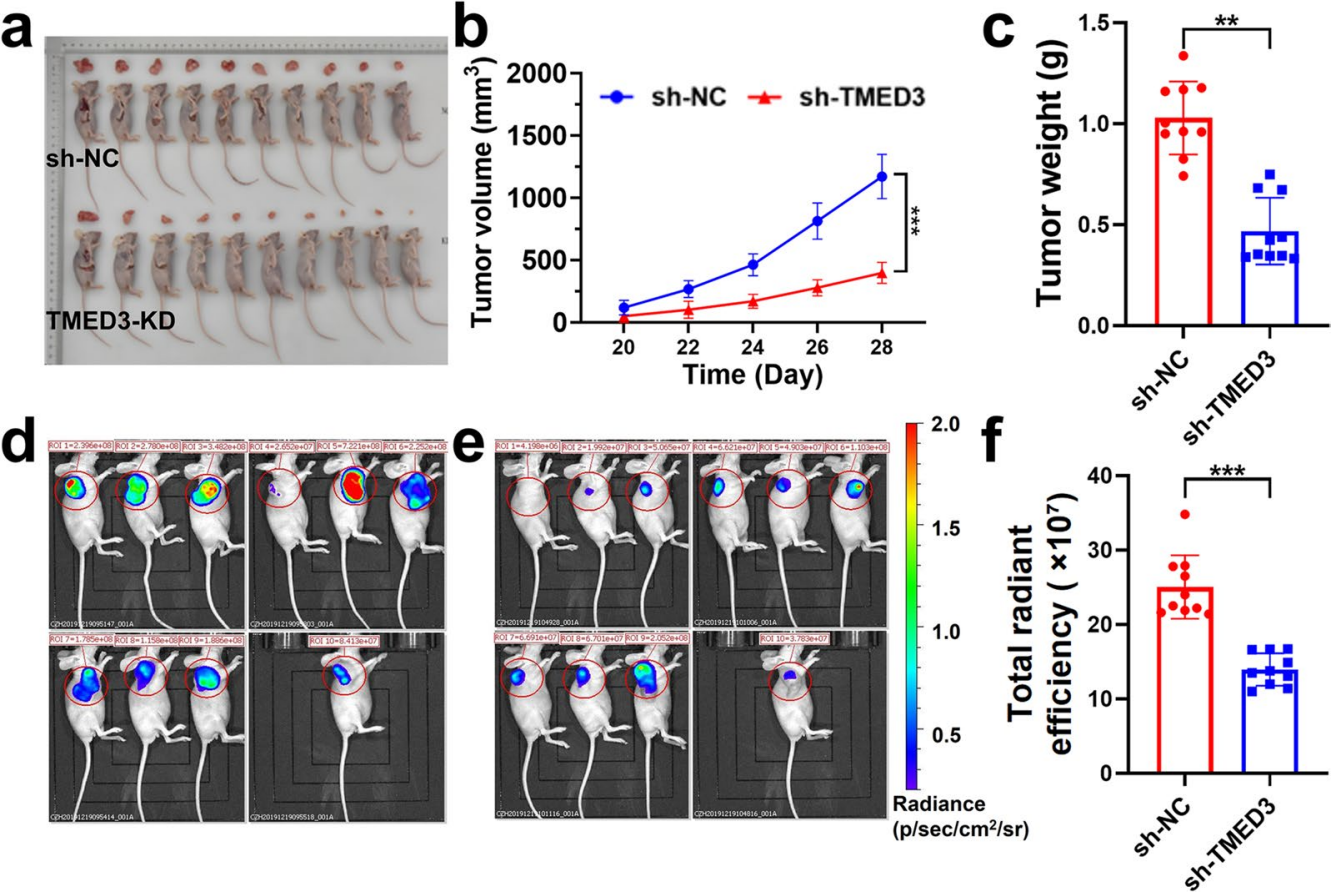
Tumorigenicity assay in nude mice after TMED3-KD transfection. **a** Mice and tumors harvested on day 28 post-tumor inoculation (*n* = 10). **b** Tumor growth curves. **c** Average tumor weight for each group. **d** Tumor fluorescence expression in the NC group. **e** Tumor fluorescence expression in the KD group. **f** Tumor fluorescence expression analysis. (*n* = 3, *P < 0.05, **P < 0.01, ***P < 0.001)

After validating the impact of TMED3 on the growth, migration, invasion, and apoptosis of GBM cells through in vitro experiments, we further investigated its role in glioma initiation and progression using in vivo models. U87 cells transduced with TMED3-KD lentivirus (KD group) or control cells (NC group) were subcutaneously injected into nude mice, with 10 mice in each group. Starting from day 20, the mice were weighed every two days, and tumor volume was measured five consecutive times. On day 28, the fluorescence expression of the tumors was measured, after which the mice were euthanized, and tumor tissues were collected for measurement and statistical analysis (Fig. 4a). The results showed that the tumor growth rate in the KD group was significantly lower than that in the NC group (Fig. 4b), and the average tumor weight in the KD group on day 28 was also significantly decreased (*P* < 0.05) (Fig. 4c). Additionally, the fluorescence intensity of tumors in the KD group was notably lower compared to the NC group (*P* < 0.05), as further confirmed by the results shown in Fig. 4d-f. These findings demonstrate that TMED3 knockdown inhibits tumorigenic effects in mice.

### Co-IP assays demonstrate that ZBTB7A is a downstream protein directly interacting with TMED3

**Fig. 5:**
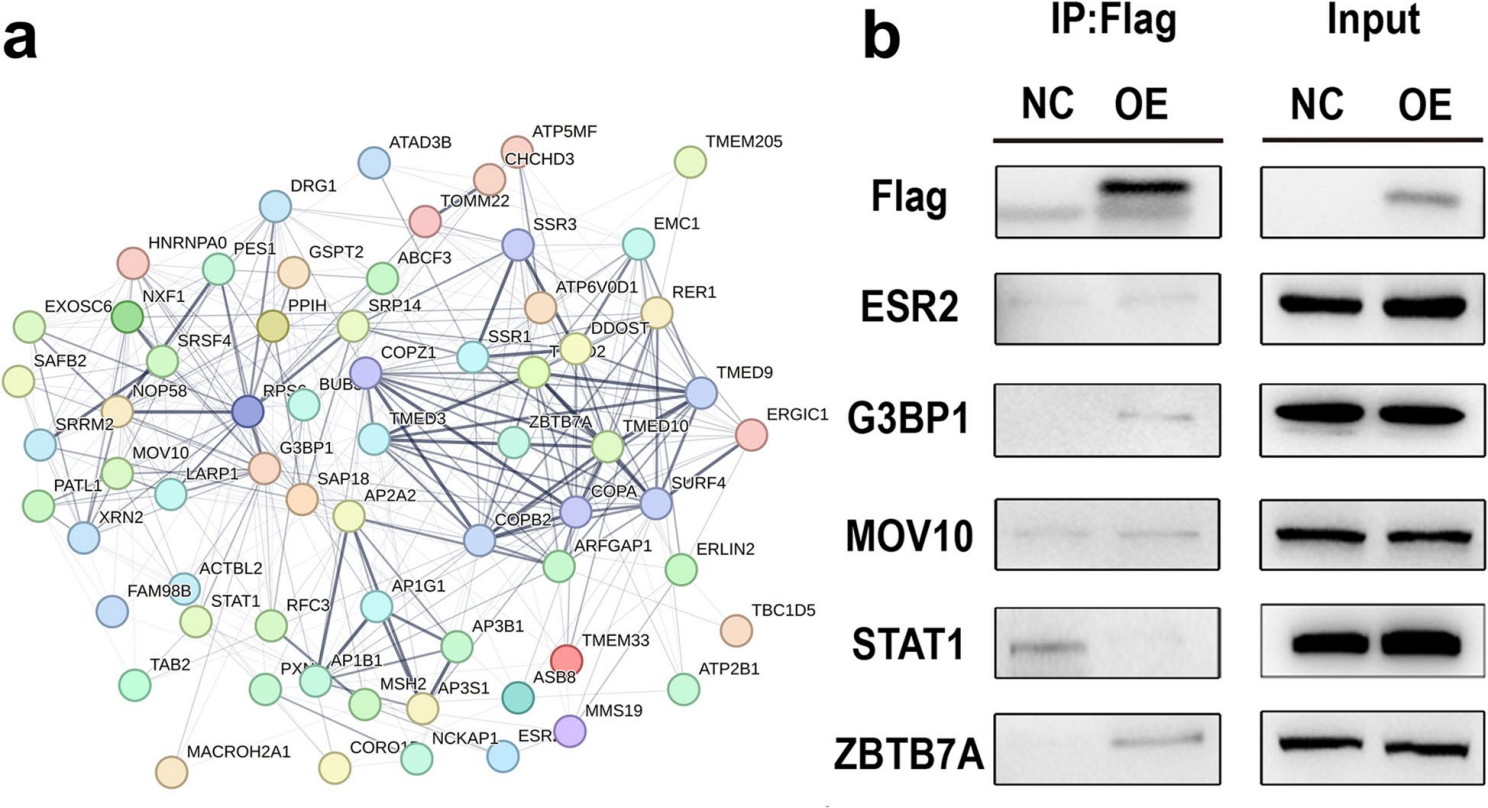
Identification of TMED3-interacting proteins. **a** Gene interaction network. **b** Co-IP validation of interacting proteins

After validating the role of TMED3 in GBM development through in vivo and in vitro experiments, we further explored downstream genes associated with TMED3 to elucidate the molecular mechanisms by which TMED3 influences GBM progression. Mass spectrometry analysis identified 1,088 proteins in the NC group and 677 proteins in the OE group, with 1088 proteins potentially interacting with the target protein (Tab S3). Using these data, we constructed a gene interaction network (Fig. 5a and S5) and selected five proteins (STAT1, G3BP1, MOV10, ESR2, and ZBTB7A) for subsequent validation.

Western blot analysis detected the presence of FLAG in both the OE and IP groups, indicating successful overexpression of 3 × FLAG-TMED3 in the target cells and its ability to bind to FLAG beads. ESR2, G3BP1, MOV10, STAT1, and ZBTB7A were detected in the input samples. In the IP samples, target bands were observed in both the NC and OE groups, but only G3BP1 and ZBTB7A showed notable increased expression in the OE group, suggesting that 3 × FLAG-TMED3 specifically captured G3BP1 and ZBTB7A. These results indicate that TMED3 interacts with these proteins, while the other proteins were not captured by 3 × FLAG-TMED3 (Fig. 5b). Therefore, G3BP1 and ZBTB7A were preliminarily identified as downstream genes of TMED3.

### ZBTB7A overexpression reverses the inhibitory effects of TMED3 knockdown

**Fig. 6:**
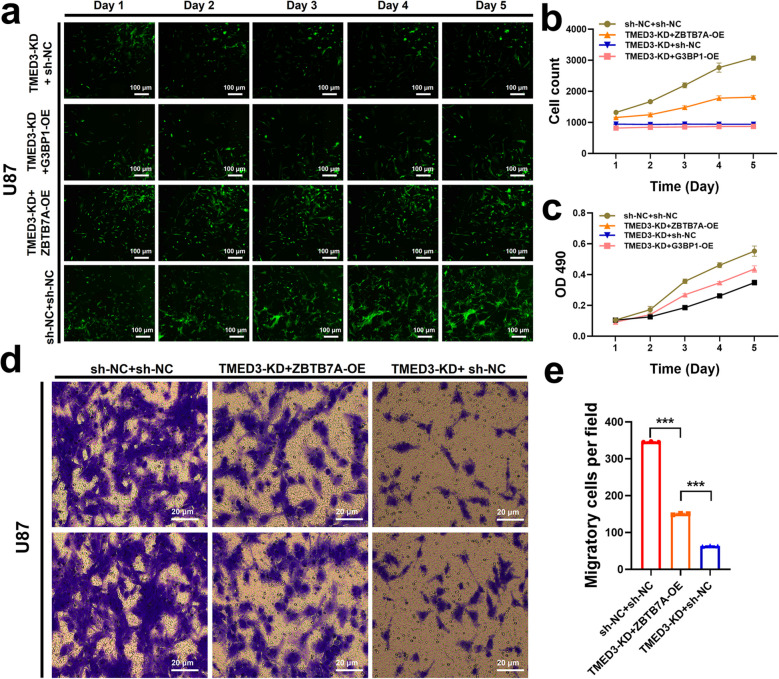
ZBTB7A reverses the inhibitory effect of TMED3. **a**,** b** Celigo cell count results and statistical analysis. **c** MTT assay results. **d**,** e** Transwell assay results and statistical analysis. (*n* = 3, *P < 0.05, **P < 0.01, ***P < 0.001)

The Celigo cell count assay revealed that compared to the sh-NC + sh-NC group, the TMED3-KD + sh-NC group exhibited a significant reduction in cell proliferation. No significant statistical difference was found between the TMED3-KD + G3BP1-OE group and the TMED3-KD + sh-NC group. However, a significant recovery in cell proliferation was observed in the TMED3-KD + ZBTB7A-OE group (Fig. 6a, b). Therefore, we selected the ZBTB7A gene for subsequent experiments, co-infected cells with ZBTB7A and TMED3-KD lentiviral plasmids, and analyzed their characteristics using MTT and transwell assays.

The MTT assay demonstrated that the cell viability in the TMED3-KD + sh-NC group was lower compared to the sh-NC + sh-NC group (*P* < 0.05), but the cell viability in the TMED3-KD + ZBTB7A-OE group was higher than in the TMED3-KD + sh-NC group (*P* < 0.05). These results indicate that overexpression of the ZBTB7A gene significantly reversed the proliferation inhibition induced by TMED3 knockdown (Fig. 6c).

The transwell assay indicated that the metastatic potential of the TMED3-KD + sh-NC group was reduced compared to the sh-NC + sh-NC group (*P* < 0.05). In contrast, the metastatic ability of the TMED3-KD + ZBTB7A-OE group was greater than that of the TMED3-KD + sh-NC group (*P* < 0.05), demonstrating that ZBTB7A overexpression reversed the inhibitory effect of TMED3 knockdown on the invasive capacity of GBM cells (Fig. 6d, e). These in vitro results preliminarily confirm that TMED3 can regulate GBM proliferation and invasion by modulating ZBTB7A.

### TMED3 knockdown reduces ZBTB7A expression

**Fig. 7:**
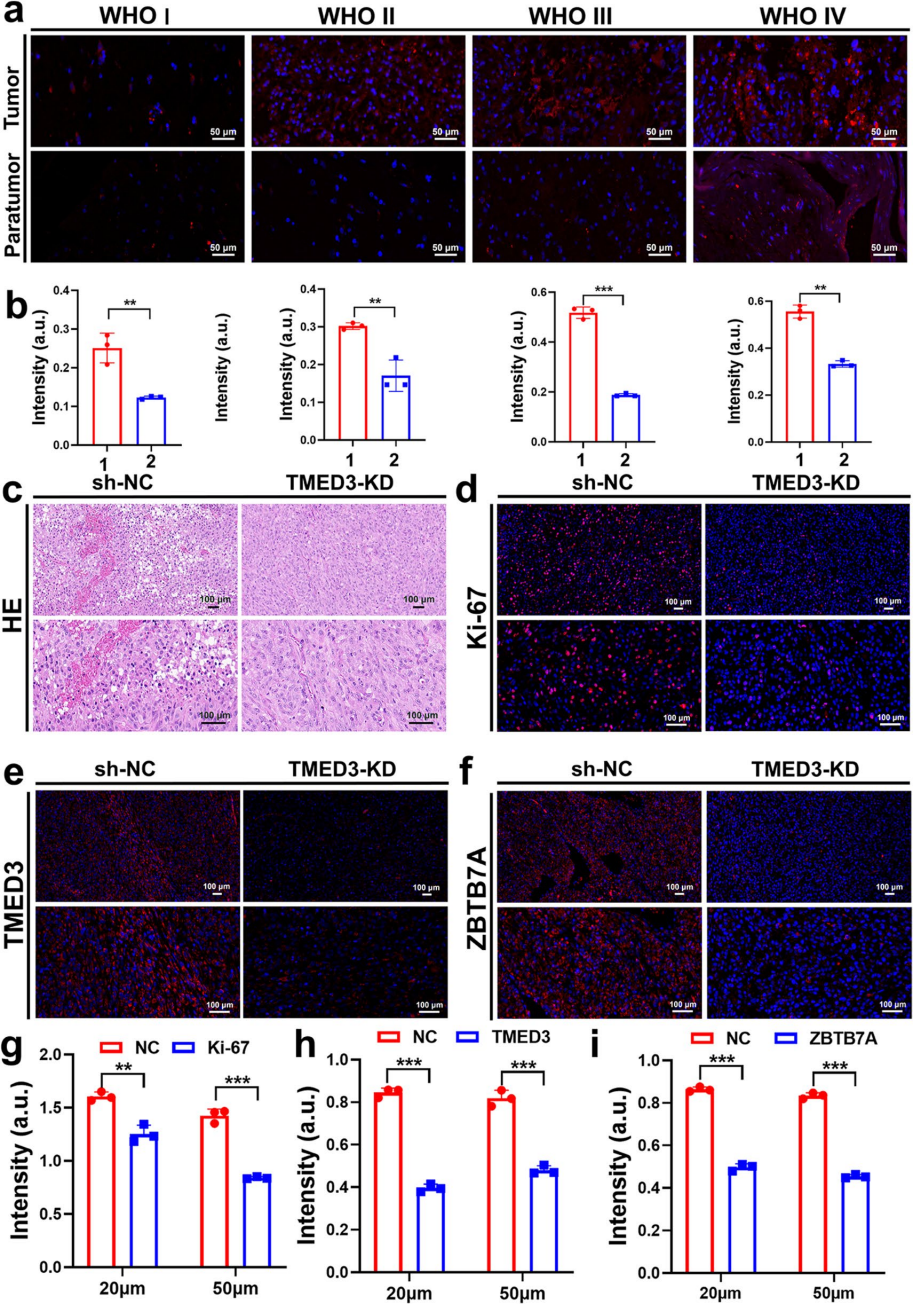
H&E staining and immunofluorescence **a**,** b** Immunofluorescence and analysis of ZBTB7A in GBM tissue samples of different grades, 1: Tumor; 2: Paratumor. **c** H&E staining of transplanted tumors. **d–i** Immunofluorescence and analysis of Ki-67 (**d**,** g**), TMED3 (**e**,** h**), and ZBTB7A (**f**,** i**) in transplanted tumors. (*n* = 3, *P < 0.05, **P < 0.01, ***P < 0.001)

After confirming the regulatory role of TMED3 on ZBTB7A in vitro, we further validated ZBTB7A expression in clinical specimens and metastatic tumors through hematoxylin and eosin (H&E) staining and immunofluorescence. As shown in Fig. 7a, b, there were significant differences in ZBTB7A expression in cancer tissues from GBM patients with different pathological grades, indicating a positive correlation between ZBTB7A expression and clinical pathological grading (Fig. 7a). H&E staining of subcutaneous tumors in mice (Fig. 7c) demonstrated that TMED3 knockout significantly reduced the malignant characteristics of the tumors. Immunofluorescence results revealed that after TMED3 knockout, the expression of Ki-67 (Fig. 7d and g), TMED3 (Fig. 7e and h), and ZBTB7A (Fig. 7f and i) were all significantly reduced. These results, supported by clinical specimens and fluorescence staining, clearly demonstrate that TMED3 regulates ZBTB7A, thereby influencing the proliferation and invasion of GBM.

## Discussion

TMED3 plays a role in the transport of proteins between the endoplasmic reticulum and the Golgi apparatus. It interacts with various transmembrane and secreted proteins and is highly expressed in various tumors, playing a dual function in either promoting or hindering tumor progression. Nevertheless, the exact function of TMED3 and the mechanisms in GBM remain unclear. ZBTB7A, also referred to as POKEMON, FBI1 and OCZF4, is part of the POZ/BTB and POK transcription factor families. It is involved in regulating key cellular processes, including proliferation, differentiation, and development [25, 26]. As an oncogene, ZBTB7A plays a critical role in tumor progression [27]. Survival studies of patients with breast cancer, prostate cancer, pancreatic cancer, bladder cancer, hematological malignancies, and hepatocellular carcinoma have shown that high expression of ZBTB7A is associated with poor prognosis [28–33]. ZBTB7A enhances metastasis in various cancers, including colorectal cancer, breast cancer and nasopharyngeal carcinoma by facilitating epithelial-mesenchymal transition, immune modulation, and altering glycolysis [32–36]. In this study, we explored the relationship between TMED3 and GBM, including its effects on tumor proliferation, invasion, and metastasis, as well as its interaction with ZBTB7A in GBM development.

Through bioinformatics analysis and single-cell sequencing of GBM and adjacent normal tissue samples, we found that TMED3 is highly expressed in GBM, and patients with low TMED3 expression exhibited better DFS and OS compared to those with high TMED3 expression. Further subgroup analysis of the samples revealed that TMED3 is a significantly differentially expressed gene in GBM, with high TMED3 expression primarily observed in GBM subgroups enriched with APOD and MAG markers. APOD, secreted by astrocytes, is involved in the transport, metabolism, and signaling of arachidonic acid, and its expression is elevated under pathological conditions [37]. MAG is an essential component of myelin protein and is detectable in GBM cells, oligodendrocytes, and neurons [38, 39]. In summary, our results confirm that TMED3 is highly expressed in GBM, and its high expression is associated with higher tumor grade and poor prognosis.

After identifying the potential role of TMED3 in GBM development, we conducted TMED3 knockdown experiments to further investigate its effects on tumor proliferation and invasion. In vitro experiments, including Celigo cell counting, apoptosis assays, and transwell assays, demonstrated that TMED3 promotes the proliferation and invasion of GBM cells. In vivo experiments, using TMED3-KD lentivirus-transfected nude mice showed a significant reduction in tumor fluorescence intensity and volume compared to controls. These in vivo and in vitro experiments confirmed the role of TMED3 in promoting GBM cell proliferation and invasion, and its inhibition can suppress tumor growth.

Having established that TMED3 accelerates the proliferation and invasion of GBM cells, we further investigated which genes or signaling axis TMED3 regulates in GBM development. Immunoprecipitation experiments identified G3BP1 and ZBTB7A as interacting proteins for further functional recovery experiments. Through Celigo cell counting assays, ZBTB7A was preliminarily identified as a positive interacting protein. Additional in vitro experiments, including MTT and transwell assays, confirmed that ZBTB7A might act as a downstream gene mediating TMED3's effects. This hypothesis was further validated through H&E staining and immunofluorescence in clinical GBM samples and nude mouse xenografts. The results confirmed that TMED3 promotes GBM cell proliferation and migration by regulating ZBTB7A.

The conclusions regarding TMED3's regulation of ZBTB7A and its impact on GBM development are significant for current research. First, AKT is a crucial regulator of several cellular processes, including metabolism, proliferation, apoptosis, and migration [40, 41]. Glycolysis, in particular, is crucial for tumor cell growth, as tumor cells primarily rely on glycolysis for energy production [42]. A study in GBM cells showed that ZBTB7A directly binds to the promoter regions of key glycolytic enzymes hexokinase 2 and lactate dehydrogenase A, transcriptionally repressing their expression [43, 44]. ZBTB7A knockdown significantly enhances aerobic glycolysis in GBM cells. Sp-1 is a transcription factor of ZBTB7A, and its overexpression increases ZBTB7A levels. Notably, Sp-1 expression is markedly elevated in human GBM tissues compared to normal tissues [45, 46]. In GBM cells, Sp-1 likely regulates the AKT signaling axis, enhancing aerobic glycolysis or promoting angiogenesis, thereby affecting tumor cell proliferation, invasion, and migration. Sp-1 deficiency reduces glucose uptake and suppresses U251 cell proliferation and invasion. TMED3 is also heavily involved in AKT regulation; inhibiting TMED3 suppresses AKT signaling, thereby inhibiting tumor growth. Conversely, AKT inhibition reduces TMED3's ability to promote tumor cell proliferation, suggesting that TMED3 functions through the AKT signaling axis [47]. In GBM, miR-1296-5p is a potent upstream miRNA of TMED3, and its suppression inhibits GBM cell proliferation, invasion, migration, and glycolysis [48, 49]. Based on this study's findings regarding TMED3's regulation of ZBTB7A, we hypothesize that AKT, as a common downstream gene in multiple signaling axes, enhances ZBTB7A expression via: (1) Increasing Sp-1 transcription, which enhances AKT expression downstream, thereby affecting tumor cell growth, invasion, and migration. (2) Increasing TMED3 and downstream ZBTB7A expression, further amplifying AKT expression. (3) Directly influencing glycolysis through ZBTB7A binding to the promoter regions of HK2 and LDHA. miR-1296-5p may serve as a shared upstream miRNA for TMED3 and Sp-1, affecting their expression and the downstream ZBTB7A signaling axis. Earlier research has demonstrated that ZBTB7A knockdown disrupts the expression of cell cycle regulatory genes and reduces cell viability in GBM cells. Sp-1 enhances ZBTB7A expression by binding to its promoter. In summary, this study identifies TMED3 as a critical regulator of ZBTB7A expression, advancing the understanding of the complex interactions among Sp-1, AKT, TMED3, and ZBTB7A. It provides important insights into the metabolic regulation and signaling network of GBM.

However, the limitations of this research must also be acknowledged. Although we elucidated the potential relationships among AKT, Sp-1, TMED3, and ZBTB7A in GBM metabolic regulation and tumor progression, further experimental validation is needed to determine whether TMED3 directly regulates ZBTB7A transcription by binding to its promoter region and whether this direct regulation is mediated in an AKT-dependent manner. Moreover, there is insufficient validation using patient samples. The hypothesis is primarily based on studies in U251 and U87 cell lines, with limited data from human GBM tissues. Employing more diverse models and clinical samples will help verify and refine the role of the TMED3-ZBTB7A signaling axis in GBM.

## Conclusions

In summary, this study identifies TMED3 as a key regulatory factor in the progression of GBM. TMED3 is significantly overexpressed in GBM, positively correlating with tumor grade and poor prognosis. Mechanistically, TMED3 promotes GBM cell proliferation, invasion, and migration by directly regulating the ZBTB7A signaling axis. These findings deepen our understanding of the molecular mechanisms of GBM, filling a gap in the regulatory network of the TMED3-ZBTB7A signaling axis, and suggest that targeting the TMED3-ZBTB7A axis could provide a promising strategy for therapeutic intervention.

## Materials and methods

### Patients and specimens

Seventeen neurosurgical patient specimens were included in this study, obtained from the Department of Neurosurgery at the First Affiliated Hospital of Anhui Medical University. All specimens contained both cancerous and adjacent non-cancerous tissues: 8 from grade IV GBM patients, 3 from grade III glioma patients, 3 from grade II glioma patients, and 3 from grade I glioma patients. All patients were newly diagnosed and had not undergone radiotherapy or chemotherapy prior to surgery. Written informed consent was obtained from all patients or their families, and the diagnosis of GBM or glioma was confirmed by experienced pathologists.

### Cell culture

All cell lines (U251, U87, A172) have undergone STR analysis to confirm their authenticity. The STR data can be found in Supplementary Information: STR reports. These cells were cultured in Dulbecco’s Modified Eagle’s Medium (DMEM, Invitrogen, CA, USA), supplemented with 10% fetal bovine serum and maintained in a thermostatic incubator (Thermo Fisher Scientific, HERAcell 150i, USA) at 37 °C with 5% CO_2_.

### Bioinformatics analysis

The TCGA database, which includes human genetic data for 33 cancer types (including glioma), was utilized for this study. We selected 529 glioma patient samples and 10 matched normal samples for multi-sample retrospective analysis at Sep 24, 2019. Initially, we analyzed the relationship between previously identified differentially expressed genes and patient survival time. Subsequently, based on data from 515 low-grade glioma (LGG) patients and 152 high-grade glioma (HGG) patients, we evaluated the differences in TMED3 expression levels across different glioma grades. The specific workflow was as follows: Glioma-related expression microarray data were downloaded from the TCGA database and divided into 529 disease samples and 10 normal samples based on barcode information. Data normalization and t-tests were performed using the Affy and Limma packages in R, and differentially expressed genes with *P* < 0.05 and |FC|≥ 2 were selected to verify whether TMED3 was a differentially expressed gene. Mann–Whitney U tests revealed significant differences in TMED3 expression between GBM and LGG patients (*P* < 0.05). Kaplan–Meier analysis (Log-Rank test) demonstrated that patients with high TMED3 expression had poorer prognosis (*P* < 0.05). These analyses revealed the expression characteristics of TMED3 in glioma and its potential associations with clinicopathological features and patient prognosis.

### Single-Cell whole transcriptome sequencing

Four glioma and four adjacent tissue samples were prepared as described in reference [38]. Tissues were cut into 1 mm^2^ pieces and digested using the Solo™ Tumor Dissociation Kit (BD Biosciences, USA) at 37 °C for 90 min. The reaction was terminated with RPMI-1640 medium, and single-cell suspensions were obtained by filtration and stored on ice until loading onto BD Rhapsody chips (BD Biosciences, USA) for single-cell transcriptome isolation. Whole transcriptome analysis based on the BD Rhapsody system was performed, and single-cell transcriptomes captured by microbeads were subjected to reverse transcription, second-strand synthesis, end repair, adapter ligation, and whole transcriptome amplification to generate double-stranded cDNA. cDNA libraries were amplified using the BD Rhapsody cDNA Kit and BD Rhapsody Targeted mRNA & AbSeq Amplification Kit with random primers and sequenced on the X Ten instrument (Illumina, USA) in PE150 mode (150 bp paired-end reads). Raw reads were processed using the BD Rhapsody Whole Transcriptome Analysis pipeline, including read quality filtering, annotation, molecular annotation, identification of putative cells, and generation of single-cell expression matrices. Low-quality read pairs were removed, and R1 reads were used to identify cell label sequences (CLs), UMIs, and poly-dT tails, while R2 reads were mapped to the GRCh37 genome using STAR (version 2.5.2b) and annotated. RSEC and DBEC algorithms were used to correct amplification biases, and second-derivative analysis distinguished putative cells from background noise, ultimately generating single-cell expression matrices. DBEC-corrected UMI counts were utilized for the subsequent clustering analysis. The raw gene expression matrices from both chips were transformed into Seurat objects using the Seurat R package. Genes expressed in fewer than 0.1% of cells and cells with mitochondrial genome UMI proportions exceeding 35% were filtered out. Data from two batches were integrated using CCA, and 12,118 cells from four patients were retained for further analysis. Gene expression data were normalized by the total UMI counts for each cell, and the 2,000 most variable genes were chosen for clustering analysis. PCA was applied for dimensionality reduction using the highly variable genes and the top 50 principal components were selected based on PC heatmaps, Jackstraw plots, and PC elbow plots. tSNE was used for further dimensionality reduction, identifying 19 clusters. Each cluster was annotated using classical markers, and the top 10 markers were plotted in heatmaps.

### Immunohistochemical fluorescence staining

Differences in TMED3 protein levels across glioma samples of different grades were detected using immunohistochemical fluorescence. Paraffin sections were prepared, immunostained, and observed under a microscope (Nikon, Eclipse Ti2, Japan). Images were analyzed and processed using ImageJ software to determine the size of positively stained areas, quantify TMED3 expression levels in different glioma grades, and generate statistical graphs.

### qPCR

Total RNA was extracted using TRIzol for TMED3 gene analysis. cDNA was obtained by reverse transcription of microRNA and mRNA. Reaction systems (20 µL) were configured according to the manufacturer’s instructions, and qPCR was performed using a two-step method (Bio-Rad, CFX96 Touch, USA). Melting curves were generated, and gene transcript levels were quantified using the 2–ΔΔCt method.

### Lentiviral vector construction, transfection, and detection

RNA interference target sequences were designed using the TMED3 gene as a template, and a lentiviral vector allowing RNA interference with the target gene was constructed, named hU6-MCS-CMV-EGFP, with the target sequence: CTCACAAGACCGTCTACTT. Single-stranded DNA oligos containing the interference sequences were synthesized, and double-stranded DNA was generated by annealing. These DNA constructs were directly ligated into digested lentiviral vectors and transferred into Escherichia coli receptor cells. Positive recombinant clones were identified by qPCR and verified by sequencing. Plasmids were extracted from confirmed clones. Lentivirus was added to cultured GBM cells at the recommended titer. Green fluorescence emitted by the lentiviral GFP element expressed in cells was observed under a fluorescence microscope (Leica, DMi8, Germany) every 8 to 12 h to assess transfection efficiency. After stable transfection, cells were selected with puromycin and re-screened. RNA was extracted, and TMED3 knockdown efficiency at the mRNA level was detected using qPCR.

### Celigo cell counting and MTT assay

The cells were resuspended and plated in triplicate in 96-well plates at a density of 1,800 cells per well, with 100 µL of medium added to each well. After cell dispersion, the plates were incubated in a culture incubator starting from day 2. The Celigo system (Nexcelom Bioscience, Celigo Image Cytometer, USA) was used to count and capture images of the cells daily for five consecutive days. The number of green fluorescent cells was calculated using a counter, and data from different groups were plotted to generate cell proliferation curves. For the MTT assay, cells were prepared in the same manner, followed by the addition of 20 µL of MTT (5 mg/mL). After 4 h of incubation, the reaction was terminated by adding 100 µL of dimethyl sulfoxide. Absorbance was measured at 490/570 nm using a microplate reader (BioTek, Synergy H1, USA), and the data were statistically analyzed.

### Apoptosis assay

Cells were resuspended in a complete culture medium and centrifuged at 1,000 rpm for 5 min in 5 mL centrifuge tubes. Subsequently, the cells were washed with pre-cooled D-Hanks (pH = 7.2–7.4) at 4 °C and once with 1 × binding buffer, followed by centrifugation at 1,000 rpm for 3 min to collect the cells. For the apoptosis assay, cells were resuspended in 200 µL of 1 × binding buffer and mixed with 10 µL of annexin V-allophycocyanin. The reaction volume was adjusted by adding an appropriate amount of 1 × binding buffer. Staining was assessed after incubation in the dark for 10–15 min at room temperature.

### Scratch assay

The cells were plated in 6-well plates with DMEM that were supplemented with 10% fetal bovine serum. After cell dispersion, a scratch was made using a scratch tool, and detached cells were removed. The medium was swapped for serum-free medium, and the cells were examined under a microscope at 0, 24, and 48 h.

### Transwell assay

Cells were seeded in 24-well plates according to predetermined groups, with 600 µL of medium containing serum added to the lower chamber. After 24 h of incubation, the medium was removed from both chambers, and non-invasive cells in the upper chamber were removed using a cotton swab. The cells were then fixed with 500 µL of paraformaldehyde for 30 min, washed several times, and counted and imaged under a microscope.

### In Vivo tumorigenicity assay

The animal experimentation protocol was evaluated and approved by the Ethics Committee of the hospital. Subcutaneous tumors were established in female nude mice (n = 10 per group) using U87 cells. Prepared cells were injected at a volume of 300 µL using a 100 µL Hamilton needle and a sterile syringe. Tumor size was measured on days 20, 22, 24, 26, and 28 post-injection. Tumor condition was assessed, and mice were weighed. On day 28, tumor size was evaluated based on fluorescence intensity using an in vivo imaging system (Perkin Elmer, Lumina LT, USA). Mice were then euthanized by cervical dislocation after an overdose injection of 2% pentobarbital sodium (0.5 mL). After fixation with 4% paraformaldehyde, the tumor specimens underwent paraffin embedding, were sectioned using a microtome, and finally processed with H&E staining for microscopic analysis.

### Lentiviral overexpression vector construction

The TMED3 gene was used as the target gene for overexpression in U87 MG cells. The 3 × FLAG tag sequence was fused to the 3'end of the TMED3 gene via qPCR, generating a 3 × FLAG-TMED3 fusion gene. The fusion gene was cloned into a lentiviral expression vector to generate the expression plasmid (p3 × FLAG-TMED3). Lentiviral particles were produced by co-transfecting the empty vector (control) and the p3 × FLAG-TMED3 plasmid along with helper plasmids. Target cells were infected with the resulting lentil-Control (NC group) and lentil-3 × FLAG-TMED3 (OE group) lentiviruses, and stable cell lines were selected using puromycin. NC and OE group cells were cultured to 80% confluence, lysed with radioimmunoprecipitation assay buffer, and sonicated on ice. Total proteins were collected by centrifugation, and protein concentration was determined using the BCA assay. The expression of 3 × FLAG-TMED3 was verified by Western blot.

### Co-IP assay

A Co-IP pre-experiment was performed using FLAG beads (Sigma, A2220) to confirm the effective enrichment of 3 × FLAG-TMED3 and the detection of Input and IP samples with FLAG antibody, following the manufacturer’s instructions. The results showed successful detection of the Flag tag in the OE group, indicating successful overexpression of 3 × FLAG-TMED3 and its recognition by Anti-Flag antibody, suitable for subsequent Co-IP experiments.

### In-gel digestion and mass spectrometry analysis

Stable NC and OE cell lines were cultured separately, and cells from 3–4 culture dishes (245 mm) with > 80% confluence were collected. After lysis with RIPA buffer, the cells were sonicated on ice and centrifuged to collect the supernatant for BCA quantification. Equal amounts of protein were subjected to Co-IP using FLAG-beads (Sigma), and the purified complexes were analyzed by SDS-PAGE and Coomassie blue staining. The results showed the presence of the Flag tag band in the OE group, indicating successful enrichment of 3 × FLAG-TMED3. Target protein bands from the SDS-PAGE gel were excised. The in-gel digestion steps included: grinding the gel slices, followed by reduction with DTT, alkylation with IAA, and trypsin digestion at 37 °C for 20 h. The resulting peptides were desalted, dried, and re-dissolved in 0.1% formic acid. Mass spectrometry analysis was performed using the Easy nLC system (Thermo Scientific) with 0.1% formic acid in water (solution A) and 80% acetonitrile/0.1% formic acid (solution B) as the mobile phases. Peptides were separated on an Accum PepMap RSLC column (50 μm × 15 cm) with a gradient from 5 to 100% solution B over 60 min at a flow rate of 300 nL/min. The Q-Exactive mass spectrometer was operated in positive ion mode, with a resolution of 70,000 for MS1 (m/z 350–1800) and 17,500 for MS2 (HCD collision energy of 27 eV). The raw data were processed using Proteome Discoverer 2.1 software, and the MASCOT 2.6 database was searched (FDR < 1%) (Tal S4). The results identified 1,088 proteins in the NC group and 677 proteins in the OE group, with 108 proteins recognized as potential interactors of TMED3.

### Bioinformatics analysis and Co-IP validation

Proteins that were specifically identified in the OE group based on mass spectrometry data were selected and analyzed through bioinformatics using the following criteria: (1) genes anticipated to interact with TMED3; (2) genes linked to cell proliferation and metastasis; (3) key genes reported in tumors. STAT1, G3BP1, MOV10, ESR2, and ZBTB7A were selected as candidate interactors for subsequent Co-IP validation.

### Functional rescue experiment

Lentiviruses containing the TMED3 target gene or selected downstream genes were used for infection experiments. The functional rescue effects of protein interactions were identified using the Celigo system and MTT assay, while the effects of interactions with downstream proteins were assessed via transwell assay. The experimental groups were as follows: sh-NC + sh-NC group: normal target cells infected with negative control virus; TMED3-KD + sh-NC group: normal target cells infected with TMED3 knockdown virus and empty control virus; TMED3-KD + ZBTB7A-OE group: normal target cells infected with TMED3 knockdown virus and ZBTB7A overexpression virus.

### Statistical analysis

Data were standardized and subjected to t-tests using the Affy and Limma packages in R, followed by filtering based on *p* < 0.05 and |FC|≥ 2. The Mann–Whitney U test was used to analyze the clinical significance of differences in TMED3 expression levels, and the relationship between TMED3 expression and clinical characteristics was analyzed using Spearman correlation. The Kaplan–Meier method (Log-Rank test) was used to evaluate the impact of TMED3 expression on patient survival. Statistical data were processed using IBM SPSS Statistics 18 and Origin 2024 software. Statistical comparisons between groups were conducted with Student's t-test, while GraphPad Prism 8.0 software was employed for generating histogram visualizations. A *p*-value threshold of 0.05 was established for determining statistical significance. ImageJ (v1.53) was used for fluorescence intensity quantification; GraphPad Prism (v9.0) and Adobe Photoshop (v2022) were used for statistical plotting.

## Supplementary Information

Supplementary Material 1.

## Data Availability

The authors confirm that the data supporting the findings of this study are available within the article.

## Abbreviations

Glioblastoma: GBM
TMED3: Transmembrane Emp24 Domain Trafficking Protein 3
Co-IP: Co-immunoprecipitation
APOD: Apolipoprotein D
MAG: Myelin-associated glycoprotein
OS: Overall Survival
DFS: Disease-Free Survival
