# Supplementary material for: Transmembrane emp24 domain-containing protein 3 promotes the malignant progression of glioma by regulating the ZBTB7A signaling axis
Source: Mol Biomed. 2025 Jun 5;6:35. doi: 10.1186/s43556-025-00274-7 (PMC12141185; doi:10.1186/s43556-025-00274-7)
Supplement: Supplementary file 1 — Supplementary Material 1. [file 43556_2025_274_MOESM1_ESM.zip › 43556_2025_274_MOESM1_ESM.docx]

**Transmembrane emp24 domain-containing protein 3 promotes the malignant progression of gliomas by regulating the ZBTB7A** **signaling axis**

**Yang Qiao^1#^, Lv Zhou^1#^, Jianyu Nie^1^, Jinshui Li^2^, Yangchun Hu^1^, Peng Gao^1^, Bingshan Wu^1*^, Hongwei Cheng^1*^, Xingliang Dai^1*^**

1. Department of Neurosurgery, the First Affiliated Hospital of Anhui Medical University, Hefei, 230001, Anhui, China

2. Department of Clinical Medicine, the Second Clinical College of Anhui Medical University, Hefei, 230032, Anhui, China

^#^ Contributed equally to this work

^*^Author to whom any correspondence should be addressed:

1. mail: daixingliang@[ahmu.edu.cn](mailto:hongwei.cheng@ahmu.edu.cn) (X. Dai); [hongwei.cheng@ahmu.edu.cn](mailto:hongwei.cheng@ahmu.edu.cn) (H. Cheng); [wubingshan@gmail.com](mailto:wubingshan@gmail.com) (B. Wu)

**Supporting information**


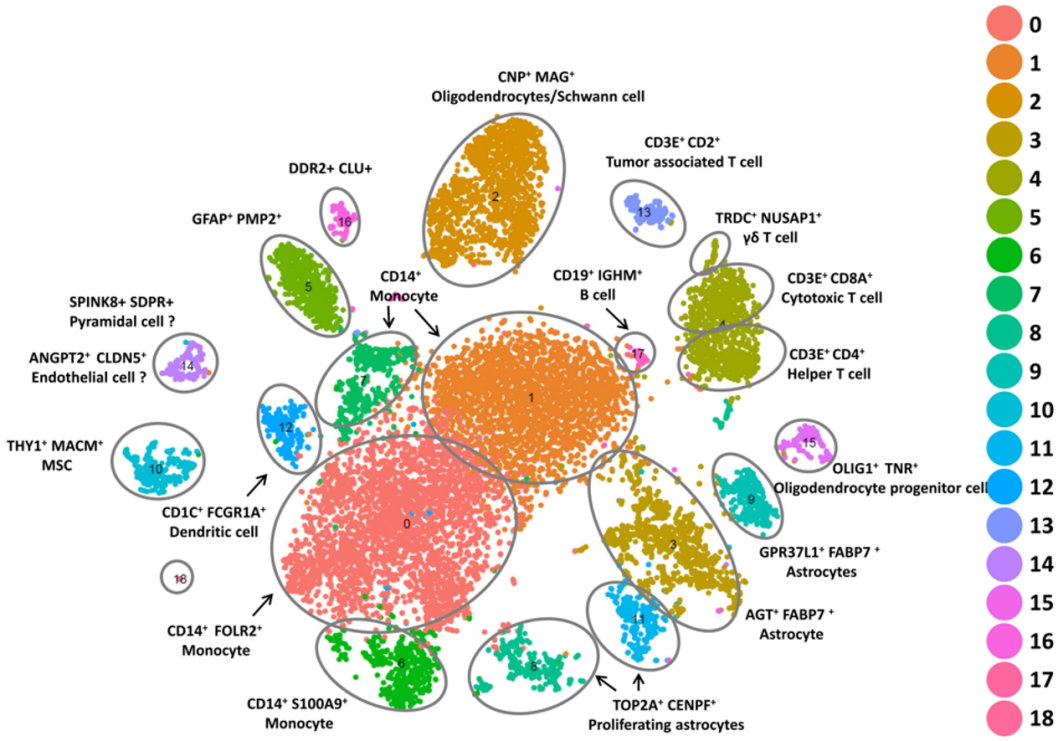


**Fig. S1** Identification for representative subpopulations.


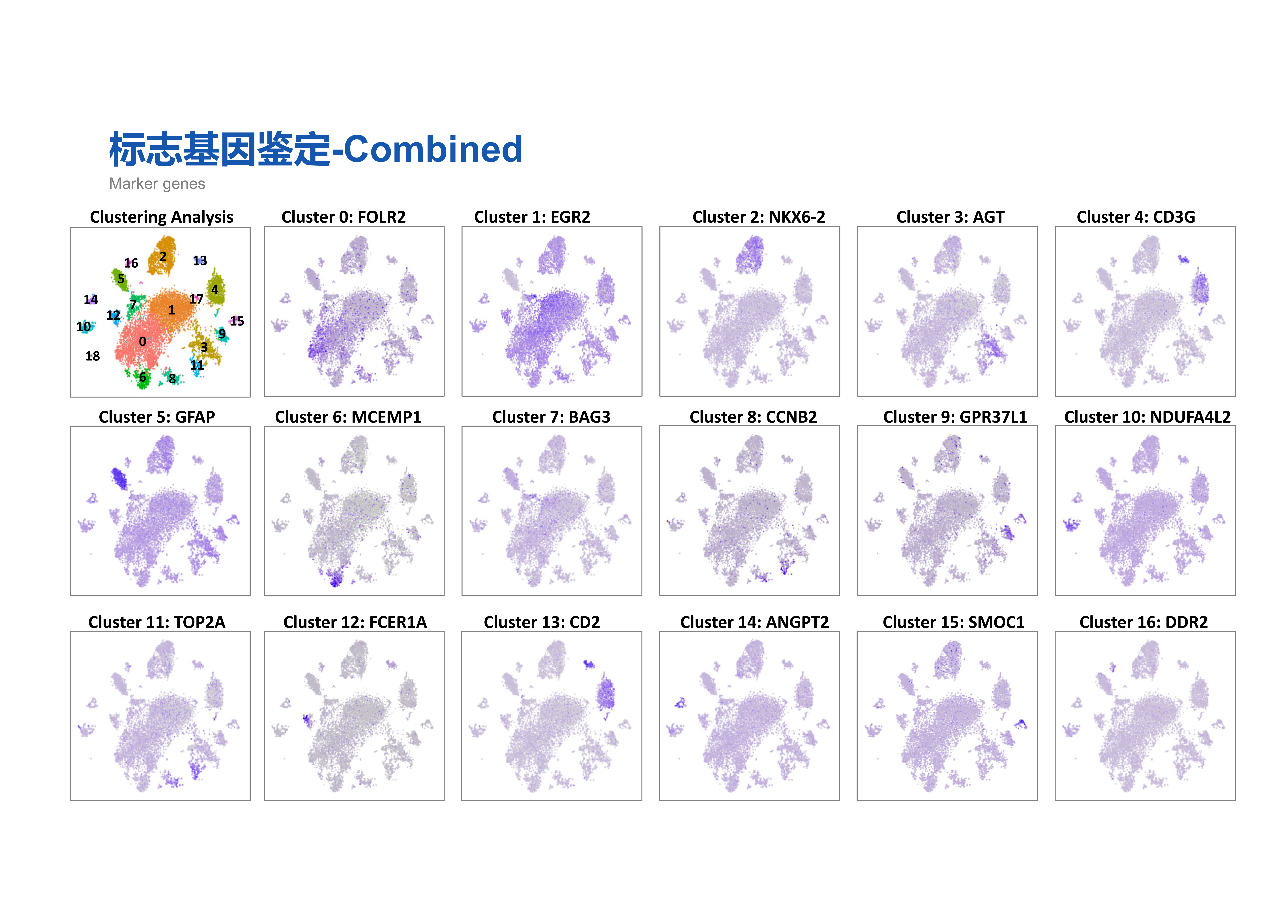


**Fig. S2** Identification of marker genes for representative subpopulations.


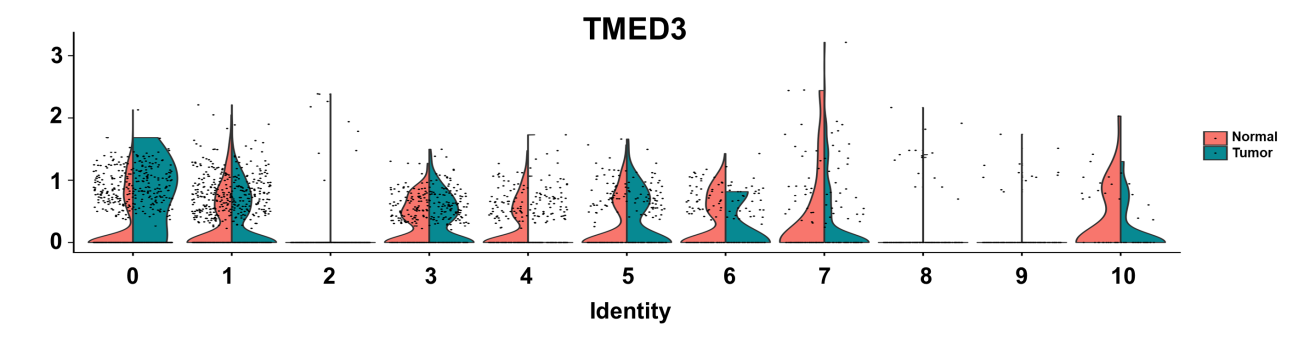


**Fig. S3** The expression level differences of TMED3 between normal and tumor tissues across different subgroups of the re-clustering analysis of glioma cell.


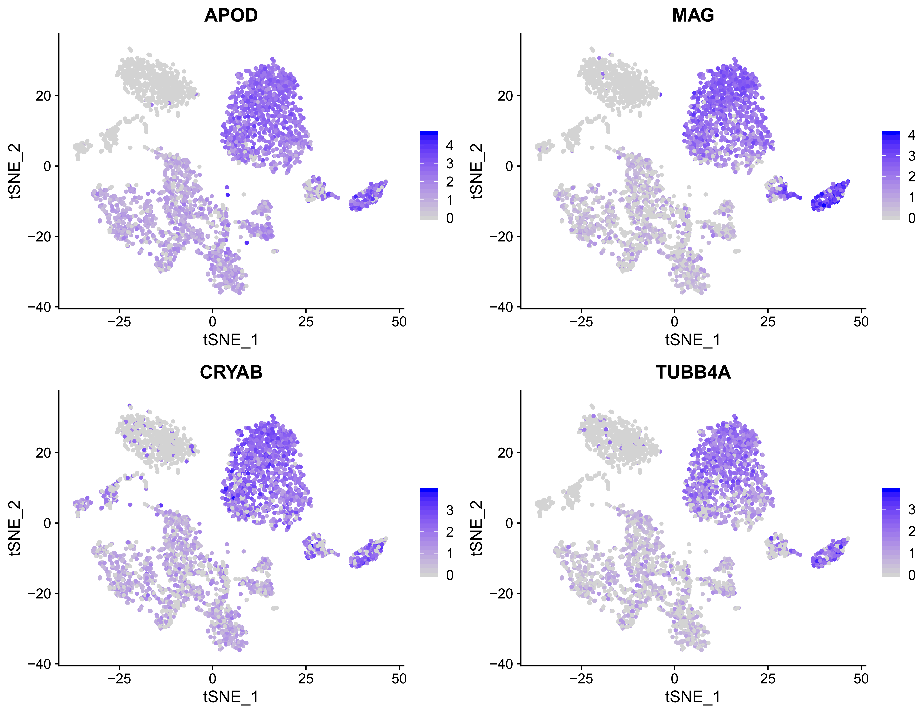


**Fig. S4** Identification of marker genes for glial subpopulation 0.


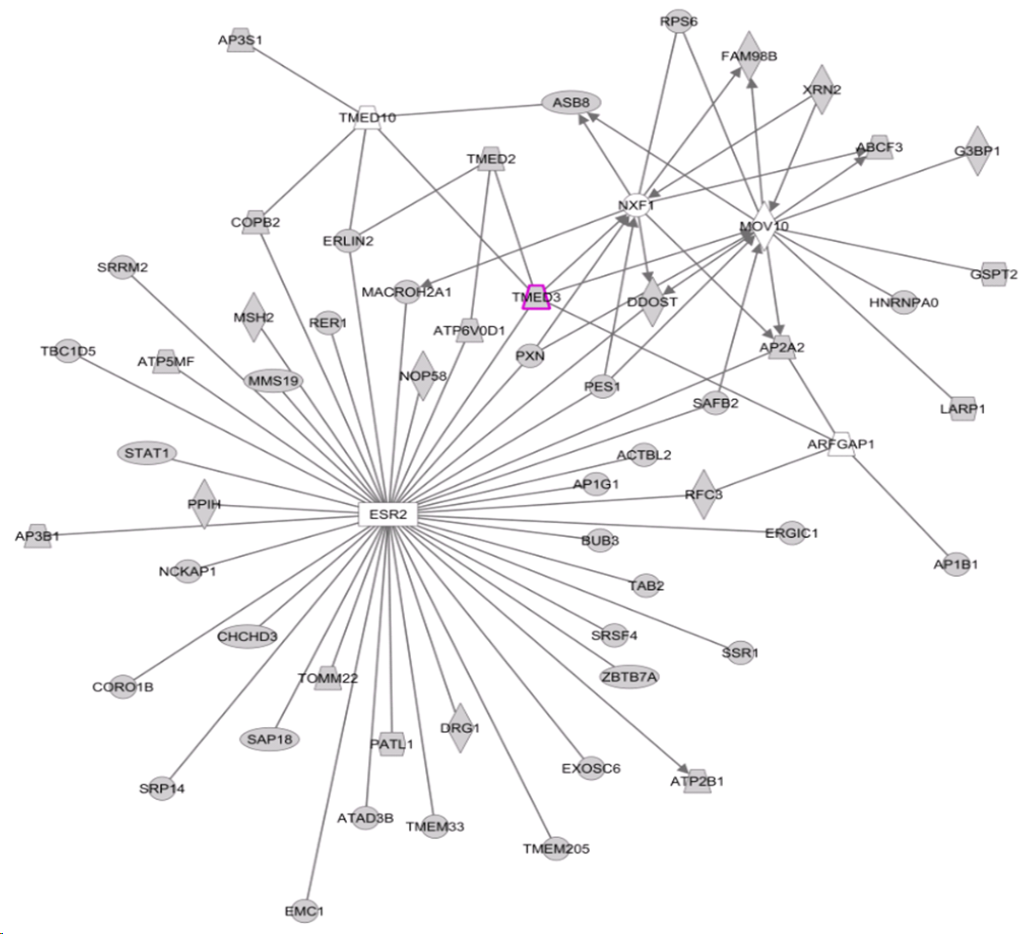


**Fig. S5** Gene-gene interaction network node plot. Gray nodes represent genes present in the original network, uncolored nodes indicate newly added genes, and purple nodes denote the target gene of interest.

**Tab S1. TMED3 expression levels in glioma tissues of different grades**

**(LGG:** low-grade glioma; **HGG:** high-grade glioma**)**

**Tab S2. The expression level of genes and the survival equivalent test of clinical data**

**Tab S3. Protein identification quantity results**

**(Database**: The name of the species used in the database; **Total spectra**: The total number of secondary mass spectra; **Spectra (PSM, Peptide Spectrum Match)**: The number of spectra matched to identified peptides; **Peptides**: The total number of identified peptides; **Protein groups**: The total number of identified protein groups)

**Tab S4. Parameters and Instructions for Processing Mass Spectrometry RAW Files**

| **Item** | **Value** |
| --- | --- |
| - **Protein Database** | - Uniprot_HomoSapiens_20367_20200226 |
| - **Enzyme** | - Trypsin |
| - **Max Missed Cleavages** | - 2 |
| - **Instrument** | - ESI-TRAP |
| - **Precursor Mass Tolerance** | - ± 10 ppm |
| - **Fragment Mass Tolerance** | - 0.05Da |
| - **Use Average Precursor Mass** | - False |
| - **Dynamic modifications** | - Oxidation (M) - Acetyl (Protein N-term) |
| - **Static modifications** | - Carbamidomethyl (C) |
| - **Database pattern** | - decoy |
| - **Peptide FDR** | - ≤0.01 |
